# A Simple Programmable Cas12a/crRNA Induced Walking System for Sensitive Methicillin-Resistant *Staphylococcus aureus* Detection via Integrated *cis*- and *trans*-Cleavage Activity

**DOI:** 10.4014/jmb.2511.11026

**Published:** 2026-01-18

**Authors:** Bo Xiao, Jie Zhang

**Affiliations:** Department of Gastrointestinal Surgery, The People’s Hospital of Nanchuan Chongqing, Chongqing City 408400, P.R. China

**Keywords:** Cas12a/crRNA, Anal fistulas, Methicillin-resistant *staphylococcus aureus*, *trans*-cleavage, AuNPs, DNA walker

## Abstract

Methicillin-resistant *Staphylococcus aureus* (MRSA) represents a serious threat to public health due to its strong antibiotic resistance, wide dissemination, and high infection rates. Rapid identification of MRSA strains is essential for accurate diagnosis and timely treatment of related infections. In this study, we propose an analytical method for MRSA that employs a hairpin-structured locker-probe to directly regulate the *trans*-cleavage activity of Cas12a. This designed locker-probe connects a target-specific aptamer to an inhibitory aptamer of the CRISPR/Cas12a system. Upon binding to the specific target, the probe undergoes a conformational change that abolishes its inhibitory effect on Cas12a. As a result, the structure-switchable probe modulates Cas12a activity in a target-dependent manner. Additionally, the sensing substrate combines a “*cis*-cleavage trigger” and a “*trans*-cleavage trigger” to integrate both *cis*- and *trans*-cleavage activities of Cas12a/crRNA within a single probe. This design significantly simplifies the probe architecture while maintaining high signal amplification efficiency. The proposed method was successfully applied to detect MRSA, achieving a detection limit as low as 2.5 CFU/ml with high specificity. By exploiting the inhibitory aptamer of Cas12a as a regulatory element for MRSA analysis, this work expands the toolbox of CRISPR/Cas12a-based methodologies and offers a promising strategy for bacterial detection.

## Introduction

Anal fistulas represent both acute and chronic conditions arising from perianal infections [1, 2]. It is estimated that approximately one-third of patients with perianal abscesses progress to develop chronic anal fistulas. Infection plays a central role in the pathogenesis of these fistulas, making the identification of causative bacterial pathogens critical for guiding therapeutic strategies and improving clinical outcomes [3]. Moreover, bacterial pathogens associated with anal fistulas pose considerable risks to global public health, as they can be transmitted through multiple routes, including bodily fluids, contaminated food, and water, thereby facilitating widespread infection. Among various pathogenic bacteria, methicillin-resistant *Staphylococcus aureus* (MRSA) presents a particularly serious threat due to its strong antibiotic resistance, broad environmental distribution, and high infection rates [4, 5]. Thus, the development of rapid and sensitive detection methods for MRSA is essential to support effective clinical diagnosis and treatment.

Conventional techniques for MRSA detection include culture-based methods [6], enzyme-linked immunosorbent assays (ELISA) [7], and polymerase chain reaction (PCR) [8]. Culture remains the gold standard owing to its high accuracy and reliability; however, it requires extended incubation periods of 7 to 14 days, demands strict sample handling and culture conditions, and carries risks of culture failure or false-negative outcomes. Although ELISA offers advantages in speed, sensitivity, and specificity, it involves complex procedures, high costs, and potential interference from cross-reactive substances. PCR provides high specificity, sensitivity, and efficiency, yet it relies on expensive instrumentation, requires intricate sample preparation (*e.g.*, cell lysis and nucleic acid extraction) is prone to false-positive results due to suboptimal sample processing or inadequate control of reaction conditions. Therefore, there is a clear need for simpler, more reliable, and highly sensitive MRSA detection technologies.

In recent years, methods based on electrochemistry [9], fluorescence [10], colorimetry [11], and Raman spectroscopy have been widely applied in bacterial identification. Among these, fluorescent biosensors using oligonucleotides have attracted considerable interest due to their operational simplicity, high stability, and non-destructive detection. These biosensors often exploit the programmability of aptamers to achieve signal conversion and cascade amplification upon target binding, typically through engineered sequences or specific structures such as hairpins or nanomaterials [12-14]. Owing to these advantages, aptamer-based fluorescence strategies have been extensively adopted in biological detection and disease diagnostics. However, systems relying solely on aptamers for signal amplification often lack sufficient specificity and sensitivity for direct MRSA detection, highlighting an area requiring further development.

In recent years, the versatility and programmability of the clustered regularly interspaced short palindromic repeats (CRISPR) and CRISPR-associated (Cas) systems have spurred extensive research in the field of molecular diagnostics [15-18]. Among these systems, Type V CRISPR/Cas12a exhibits unique and potent collateral cleavage activity toward non-target single-stranded DNA (ssDNA) [19, 20]. This property has been widely exploited to develop a variety of signal amplification platforms by converting target recognition into highly efficient *trans*-cleavage of ssDNA reporters, with Cas12a achieving a turnover rate of approximately 17 events per second. In particular, the Cas12a/crRNA complex has been successfully applied for specific detection of MRSA genomic material through integrated cell lysis and nucleic acid extraction [21, 22]. These detection mechanisms rely fundamentally on full complementarity between the target DNA (double-stranded or single-stranded) and the spacer region of the CRISPR RNA (crRNA), which activates the RuvC catalytic domain of Cas12a to cleave both the target DNA (in *cis*) and nearby ssDNA reporter molecules (in *trans*). Nevertheless, the development of new strategies to directly modulate the *trans*-cleavage activity of Cas12a could further broaden its utility in bio-sensing.

Inspired by these advances, we aimed to construct a fluorescence-based assay for direct detection of methicillin resistance in MRSA using a programmable Cas12a/crRNA complex in conjunction with a DNA walking system. Central to this design was a rationally engineered locker-probe that contained both a target-binding sequence and an inhibitory aptamer for Cas12a. In the absence of the target, the locker-probe adopts an active flap structure, enabling its core nucleotide region to occupy the binding site of the Cas12a/crRNA complex on a double-stranded DNA substrate. This interaction facilitates duplex formation with crRNA and markedly suppresses the catalytic activity of Cas12a (resulting in a “locked” Cas12a/crRNA complex). Upon introduction of the target bacteria, the locker-probe undergoes a conformational shift to an inactive state, which prevents it from interacting with the Cas12a/crRNA complex, thereby restoring the complex’s ability to bind dsDNA. A key feature of this system is the integration of both the *trans*-cleavage trigger and *cis*-cleavage activity into a single “substrate” probe, which simplifies the probe architecture without compromising signal amplification efficiency. As a result, we established a sensitive and simple approach for direct analysis of methicillin resistance in MRSA. This method employs aptamers that specifically recognize PBP2a, a protein encoded by the *mecA* gene that confers methicillin resistance [10]. The practical value of this assay was confirmed through its application in MRSA detection, where it demonstrated high sensitivity and specificity. Moreover, we verified that the proposed strategy performs reliably in complex biological matrices, yielding satisfactory detection results. Notably, the developed assay is culture-free and operates without a pre-enrichment step, allowing for rapid and direct detection of MRSA in clinical samples.

## Experimental Section

### Materials and Reagents

All oligonucleotides used in this study (Table S1) were synthesized by Sangon Biotech Co., Ltd. (China). AuNPs were obtained from Nanjing Dongna Biotechnology Co., Ltd. (China). DNA polymerase was obtained from New England Biolabs (USA). Diethylpyrocarbonate (DEPC) water and 10× TBE buffer were supplied by Sangon Biotech Co., Ltd. The Cas12a protein (10 U/L) was sourced from Thermo Fisher Scientific Inc. (USA). All solutions were prepared using DEPC water. Fluorescence emission spectra were recorded on a microplate reader (BioTek Instrument, USA) using a transparent 384-well microplate (Fluotrac 200, Germany). Data were expressed as mean ± standard deviations, *n* = 3 technical replicates.

### Methodology for MRSA Detection

The detection of MRSA was performed in two main steps: target recycling and Cas12a/crRNA-mediated cleavage. In the target recycling step, a 10 μl sample containing different concentrations of MRSA was mixed with 10 μl of the programmable Cas12a/crRNA@AuNPs complex, 0.7 U/μl DNA polymerase, and 1× NEBuffer 3.1, followed by incubation at room temperature for 30 min. Subsequently, 20 nM Cas12a and 10 nM crRNA were introduced, bringing the total reaction volume to 18 μl. This mixture was further incubated for 30 min at room temperature. Finally, the resulting solution was transferred into a black 384-well microplate, and fluorescence readings were taken with a microplate reader using an excitation wavelength of 670 nm. Optimization of experimental parameters follows the same procedures. The concentration of MRSA used for determining experimental parameters was 10^3^ CFU/ml.

## Results and Discussion

### Design of the Programmable CRISPR/Cas12a Induced DNA Walker for MRSA Detection

**Fig. 1:**
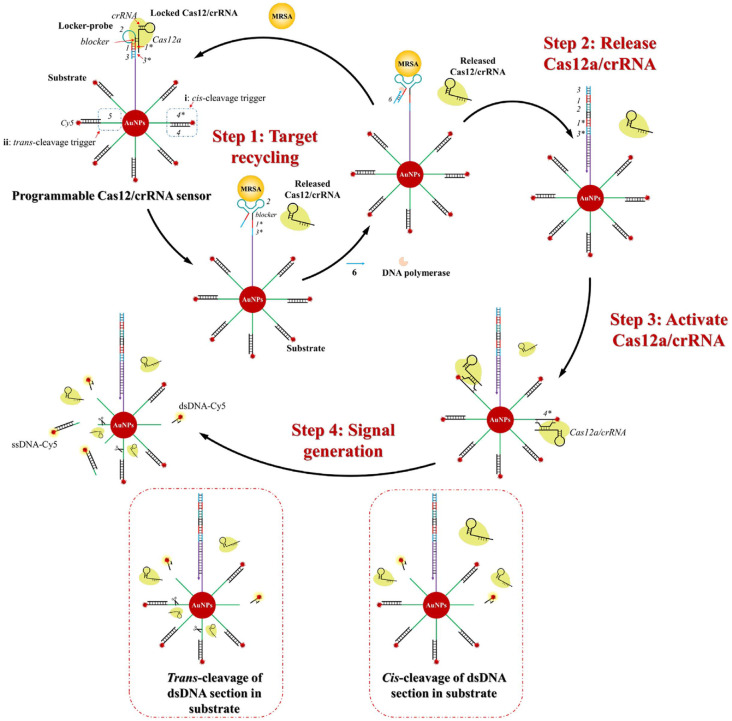
The working principle of the proposed method based on programmable CRISPR/Cas12a for the bio-sensing application.

This method employs an allosteric hairpin probe (locker-probe) that enables detection of the PBP2a protein by converting target recognition into Cas12a cleavage activity (Fig. 1). The locker-probe consists of several functional domains: sequence “2” contains PBP2a-specific aptamers [10, 23] for direct target binding; the “blocker” sequence serves as the seed region for crRNA hybridization; sequence “1*” includes a protospacer adjacent motif (PAM, 5′-NTT-3′) for Cas12a interaction; and sequences 3/“3*” form a stem structure that stabilizes the hairpin conformation. Additionally, the locker-probe is immobilized on the surface of gold nanoparticles (AuNPs). A separate double-stranded DNA probe, designated as the “substrate,” is constructed by hybridizing two complementary sequences. This substrate incorporates two functional elements: a dsDNA segment that triggers the *cis*-cleavage activity of the Cas12a/crRNA complex, and an adjacent ssDNA segment susceptible to *trans*-cleavage by the same complex. The terminus of the substrate is labeled with a Cy5 fluorophore, which becomes quenched upon adsorption onto the AuNPs surface. By integrating both *cis*- and *trans*-cleavage triggers into a single molecular entity, this substrate design simplifies the probe architecture without compromising signal amplification efficiency. In the absence of the target, the locker-probe occupies the substrate-binding site of Cas12a/crRNA, preventing the enzyme from recognizing the dsDNA segment of the substrate and thus maintaining Cas12a in an inactive (“OFF”) state. Upon introduction of the target, the “2” domain of the locker-probe binds specifically to PBP2a, causing a conformational change that separates the seed sequence from the PAM region and exposes the “3” segment. This releases the Cas12a/crRNA complex, enabling it to bind and cleave the *cis*-activation region of the substrate. Concurrently, a primer sequence (“6”) binds to the exposed “3” domain and initiates a polymerase-mediated extension reaction. This step displaces the target from the aptamer region, allowing it to participate in another cycle of Cas12a activation, thereby enabling target recycling amplification. Once activated, Cas12a cleaves both the *cis*-activating dsDNA segment and the *trans*-activating ssDNA region of the substrate, leading to the release of the Cy5 fluorophore from the AuNP surface. The resulting recovery of Cy5 fluorescence is proportional to the concentration of MRSA-derived PBP2a present in the sample.

### Loading the Substrate and Locker-Probe on the Surface of AuNPs

**Fig. 2:**
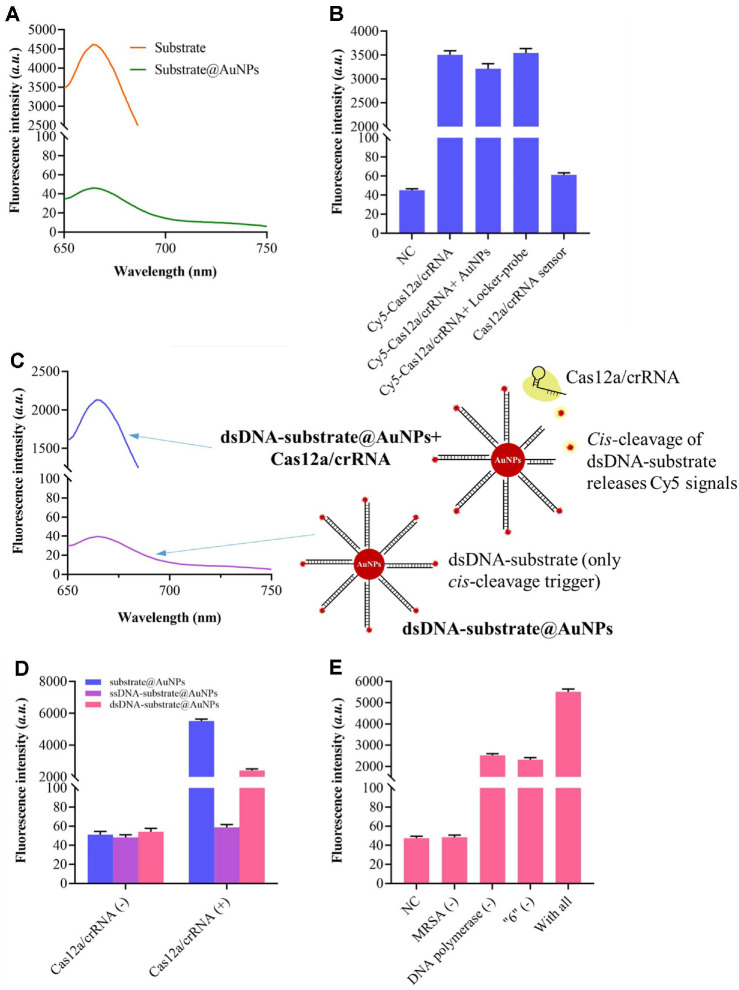
Construction of the programmable Cas12a/crRNA biosensor and feasibility analysis. (**A**) Fluorescence spectrum of the substrate before and after being loaded on the AuNPs surface. (**B**) Fluorescence intensity of the Cy5 labeled Cas12a/crRNA complex during the construction of the programmable Cas12a/crRNA complex. (**C**) Fluorescence spectrum of the dsDNA-substrate@AuNPs when Cas12a/crRNA is mixed. (**D**) Fluorescence intensity of the dsDNAsubstrate@ AuNPs, ssDNA-substrate@AuNPs, and substrate@AuNPs when Cas12a/crRNA is mixed. (**E**) Fluorescence intensity of the sensor when essential components existed or not.

The fluorescence signals of Cy5 were measured and compared to evaluate the substrate loading efficiency on the surface of AuNPs. As shown in Fig. 2A, a significant reduction in fluorescence intensity was observed after the Cy5-labeled substrate was immobilized onto the AuNPs, which can be attributed to fluorescence quenching by the AuNPs. The successful assembly of the programmable Cas12a/crRNA sensor was further confirmed in a similar manner. In this experiment, the terminal end of the crRNA was conjugated with a Cy5 label. According to Fig. 2B, the fluorescence intensity remained high prior to immobilizing the locker probe and Cas12a/crRNA complex onto the AuNPs. However, when Cas12a/crRNA was introduced to the AuNPs in the absence of the locker probe, only negligible changes in fluorescence intensity were detected, indicating poor adsorption of the complex to the AuNPs surface. A pronounced decrease in fluorescence was observed only when both the locker probe and Cas12a/crRNA were present, confirming the successful construction of the programmable Cas12a/crRNA sensor.

We next examined the *cis*-cleavage activity of CRISPR/Cas12a using a dsDNA substrate labeled with Cy5 at its terminus. This configuration maintains the intrinsic *cis*-cleavage trigger of the substrate, allowing assessment of the dsDNA recognition capability of the released Cas12a/crRNA complex. The results demonstrated that the target MRSA induced a significant increase in fluorescence, indicating that target recognition by the locker probe leads to the release of Cas12a/crRNA, which subsequently recognizes and cleaves the dsDNA substrate, thereby generating a fluorescent signal (Fig. 2C). Subsequently, the *trans*-cleavage activity of the Cas12a/crRNA complex was evaluated by comparing the fluorescence intensities of sensing systems employing either a dsDNA substrate or an ssDNA substrate. The ssDNA substrate, terminally labeled with Cy5, is cleaved only upon activation of Cas12a/crRNA by dsDNA. As depicted in Fig. 2D, a substantially enhanced fluorescence signal was detected in the system with the dsDNA substrate in the presence of Cas12a/crRNA, whereas the signal remained low with the ssDNA substrate. These results indicate that only the dsDNA substrate triggers the *trans*-cleavage activity of Cas12a/crRNA. The significantly higher fluorescence intensity in the dsDNA-based system further suggests that *trans*-cleavage induces additional substrate turnover, thereby improving the sensitivity of the assay.

Finally, the performance of the reaction systems was assessed using fluorescence spectroscopy. As illustrated in Fig. 2E, negligible fluorescence was detected in the absence of MRSA compared to the negative control. In contrast, the addition of MRSA and DNA polymerase triggered a target-specific structural rearrangement of the locker probe, yielding a strong fluorescence signal with an activation efficiency of up to 70%. These findings indicate that the catalytic activity of Cas12a can be effectively regulated by conformational changes in the locker probe.

### Optimization of Experimental Parameters

**Fig. 3:**
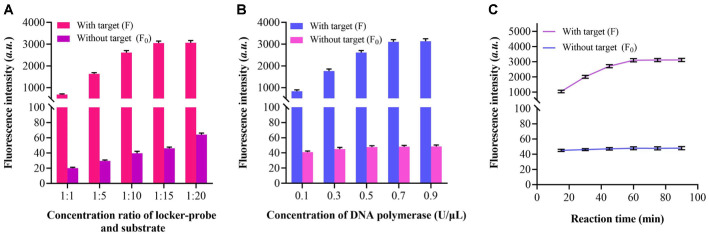
Optimization of experimental parameters. (**A**) Fluorescence signals in the presence (F) and absence (F_0_) of the target at different concentration ratios of the locker probe to the substrate. (**B**) Variation in fluorescence intensity with (F) and without (F_0_) the target under different DNA polymerase concentrations. (**C**) Fluorescence readings with (F) and without (F_0_) the target over different reaction durations.

To achieve optimal efficiency in MRSA detection, key experimental parameters were systematically optimized: the molar ratio of the locker probe to the substrate, the amount of DNA polymerase, and the reaction time. Initially, the molar ratio between the locker probe and the substrate was evaluated. As shown in Fig. 3A, as the molar ratio increases from 1:1 to 1:20, the background signal rises progressively due to the accumulation of unquenched substrate remaining in the system. The fluorescence intensity increases steadily from a ratio of 1:1 to 1:15, after which it reaches a plateau despite further increases in the molar ratio. Based on the variation in the F/F_0_ ratio, a molar ratio of 1:15 was selected for subsequent experiments.

The target recycling efficiency is closely related to the amount of DNA polymerase, which was also optimized in this study. Fig. 3B shows that as the concentration of DNA polymerase increases from 0.1 U/μl to 0.7 U/μl, the fluorescence signals rise markedly. However, further increases in polymerase concentration do not lead to additional signal enhancement. Meanwhile, background signals remain largely unchanged across the tested concentrations. Therefore, 0.7 U/μl of DNA polymerase was chosen for further assays.

Reaction time, which is crucial parameter determining the signal amplification cycles and amplification efficiency, was also investigated to achieve both rapid and stable detection. According to Fig. 3C, the relative fluorescence intensity increases significantly when the reaction time is extended from 30 to 60 min, after which it stabilizes between 60 and 90 min. Throughout this period, background signals remain relatively constant. A reaction time of 60 min was thus adopted to achieve the desired detection performance.

### Performance of the Programmable Cas12a/crRNA Biosensor

**Fig. 4:**
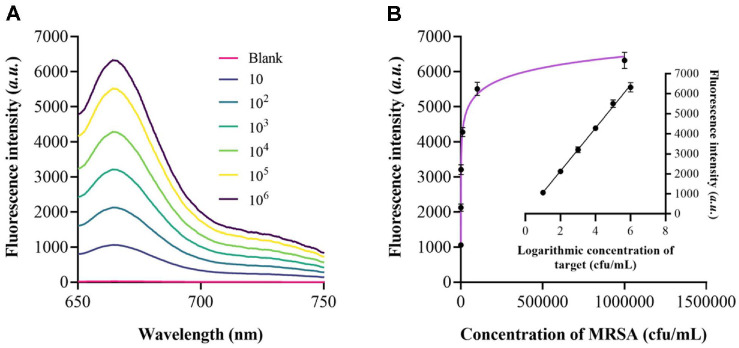
Biosensor sensitivity for MRSA detection. (**A**) Fluorescence intensity measured across a gradient of MRSA concentrations. (**B**) Corresponding calibration curve demonstrating the linear dependence of the signal on bacterial concentration.

Under the optimized reaction conditions, the sensing system was subsequently applied to detect MRSA A series of MRSA concentrations were tested, and the corresponding fluorescence spectra were acquired using a fluorescence spectrophotometer. As shown in Fig. 4A, the variation in fluorescence intensity of the biosensor was recorded upon the introduction of different MRSA concentrations. Fig. 4B depicts the relationship between MRSA concentration and the fluorescence intensity change measured at 670 nm. A marked increase in fluorescence intensity was observed as MRSA concentrations ranged from 10 to 10^6^ CFU/ml, beyond which the signal reached a plateau. The inset in Fig. 4B presents the calibration curve for MRSA quantification. A strong linear relationship (R^2^ = 0.9954) was obtained, described by the equation F = 1072 × lgC – 1.338, where F represents fluorescence intensity and C corresponds to MRSA concentration. Based on a signal-to-noise ratio (S/N) of 3, the detection limit was calculated to be 2.5 CFU/ml.

### Specificity and Practicability Analysis of the Programmable Cas12a/crRNA Biosensor

**Fig. 5:**
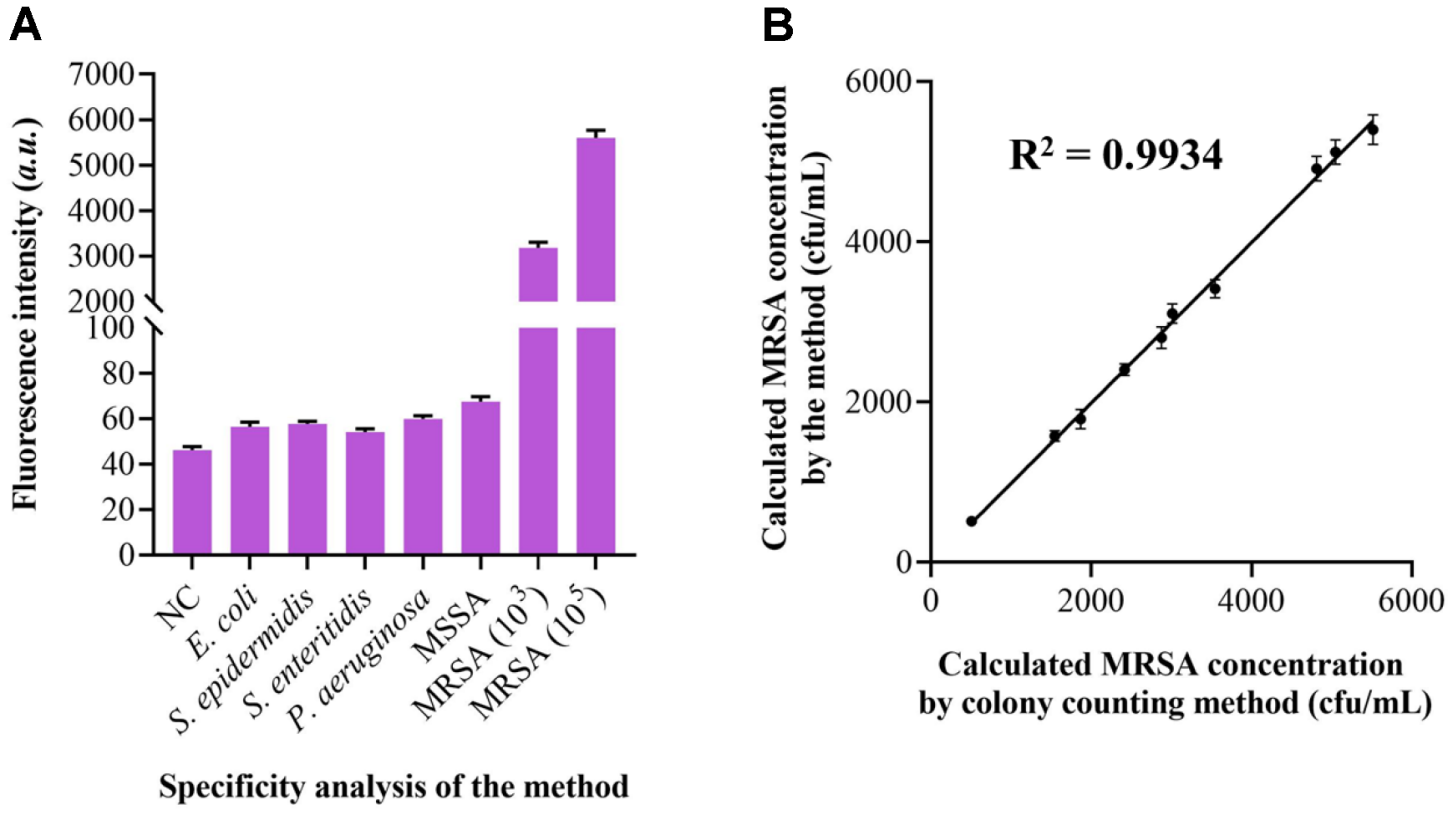
Analysis of the specificity and practical applicability. (**A**) Fluorescent signals obtained for MRSA detection along with those from potential interfering bacterial species. (**B**) Correlation of MRSA concentrations estimated by the proposed approach with results from the conventional plate counting method.

Furthermore, the analytical specificity was evaluated using a panel of prevalent bacterial strains, including MRSA, methicillin-susceptible *Staphylococcus aureus* (MSSA), methicillin-susceptible *S. aureus* (MSSA), *Escherichia coli* (*E. coli*), *Staphylococcus epidermidis* (*S. epidermidis*), *Pseudomonas aeruginosa* (*P. aeruginosa*) and *Salmonella enteritidis* (*S. enteritidis*). As illustrated in Fig. 5A, at equivalent bacterial concentrations, only MRSA induced a significant shift in the characteristic fluorescence intensity, while the other tested microorganisms produced minimal changes. This outcome confirms the high specificity of the method for MRSA detection, which can be largely attributed to the sequence-specific recognition inherent to the CRISPR/Cas12a system. The combination of exceptional sensitivity, specificity, operational simplicity, and visual readout underscores the potential of this approach in point-of-care testing for bacterial resistance. The practical applicability and accuracy of the programmable Cas12a/crRNA biosensor were further assessed using spiked MRSA samples. For comparative validation, the conventional colony counting method was employed as a reference. The results showed strong consistency between the two techniques, with a high correlation coefficient of 0.9934 (Fig. 5B), thereby verifying the reliability and precision of our bio-sensing strategy in real-sample scenarios. Additionally, recovery experiments were conducted using constructed serum samples to evaluate the method’s performance in complex biological matrices. The recovery rates ranged between 97.3% and 104.3%, indicating high stability and minimal matrix interference in clinical serum samples.

## Conclusion

This study reports the development of a fluorescent biosensor for direct detection of MRSA using a programmable Cas12a/crRNA complex. A key innovation of this approach is the design of a structure-switchable locker-probe that connects a target-specific aptamer to an inhibitory aptamer for Cas12a, enabling efficient regulation of CRISPR/Cas12a activity to identify the PBP2a protein present on the MRSA surface. Moreover, the substrate combines a “*cis*-*trans*-cleavage trigger” and a “*trans*-cleavage trigger” into a single probe, integrating both *cis*- and *trans*-cleavage functionalities of Cas12a/crRNA. This consolidation simplifies the probe architecture while maintaining comparable signal amplification efficiency. The designed programmable Cas12a/crRNA complex offers several notable advantages compared with former ones (Table S2): i) the locker-probe enhances the complex’s utility in MRSA detection; ii) combining *trans*- and *cis*-cleavage substrates into one probe simplifies experimental procedures; and iii) the sensing platform can be easily repurposed to detect other bacterial species by modifying the aptamer region within the locker-probe. Validation using various complex samples confirmed the reliability and broad applicability of this MRSA detection strategy, particularly for clinical diagnostics of bacterial infections. It should be noted that, rather than using actual clinical specimens, our validation relied on constructed blood samples to simulate clinical conditions. While this approach demonstrates the potential diagnostic utility of the method, further studies with real clinical samples will be necessary to fully establish its practical applicability in clinical settings. We anticipate that this method will inspire further development of innovative aptamer-based tools for CRISPR/Cas systems and bio-sensing applications.

## Supplemental Materials

Supplementary data for this paper are available on-line only at http://jmb.or.kr.
