# Supplementary material for: A Simple Programmable Cas12a/crRNA Induced Walking System for Sensitive Methicillin-Resistant Staphylococcus aureus Detection via Integrated cis- and trans-Cleavage Activity
Source: J Microbiol Biotechnol. 2026 Jan 18;36:e2511026. doi: 10.4014/jmb.2511.11026 (PMC12828325; doi:10.4014/jmb.2511.11026)
Supplement: Supplementary file 1 [file jmb-36-e2511026-supple.pdf]

J. Microbiol. Biotechnol. 2026. 36: e2511026

https://doi.org/10.4014/jmb.2511.11026

Supplementary Tables

Supplemented experimental section

Source of bacteria

Salmonella enteritidis (CMCC(B) 50335) was provided by China Center for Type Culture Collection. Methicillin-resistant S. aureus (MRSA, ATCC 43300), Salmonella typhimurium (ATCC 14028), Bacillus subtilis (ATCC 6633), and Escherichia coli (E. coli, ATCC 8739) were purchased from China General Microbiological Culture Collection Center. The ultrapure water (18 mΩ) used in all solutions comes from the Millipore water purification system.

Preparation of Bacteria and Identification Structure.

The bacteria were grown in LB medium at 37 °C for 24 h and then the bacterial concentration was quantified by optical density. Subsequently, it was centrifuged at 8000 rpm for 10 min and dispersed in PBS buffer, repeated three times for standby. The hairpin structure was obtained by annealing substrate and locker-probe (6 μL, 10 μM) in 10 mM TE buffer containing 5 mM Mg^{2+} at 90 °C for 10 min and then cooling to room temperature, respectively.

Preparation of probe-functionalized AuNPs

100 μM probes heated at 90 °C for 3 min and then cooled at 4 °C for 1 h. The received T-probes (5′-thiol-modified probes) to be in a disulfide form that mercaptohexanol group protects it. DTT as a reducing agent disrupt any disulfide bonds (-S-S-) and reduce them to free SH groups that they ready to interaction with gold surface and adsorbed on AuNPs. 10 μL DTT solution (1.0 N) and 50 μL (50 μM) probes were mixed using a vortex mixer. To ensure that all disulfide groups are fully split, the mixture kept at room temperature for 1.5 h. Via extracting with ethyl acetate 3 times using 50 μL per extraction, unfavorable thiol segments and excess DTT remove from the thiol-modified probe mixture. After vortexing the mixture, the upper layer is discarded. The lower layer is T-probes that ready to react directly to gold nanoparticles. Afterward, 40 μL AuNPs and 10 μL 0.5 μM T-probes were incubated together for overnight. The T-Apts were adsorbed on the surface of AuNPs through firm covalent bond between SH and gold.

Table S1. Sequence information for oligonucleotides was used in this study

Title		Sequences (5' to 3')	Labeling
Locker-probe		CGT TTG ACC TGG GGG AGC ATT GCG GAG GAA GGT GAG TAC AAA CG	
crRNA		AAU AAU UUC UAC UAA GUG UAG AUC UCA GCG CCA ACU AGA UCGA	
Substrate			
	dsDNA	ACT TGT CGA TCT AGT TGG CGC TGA	

		GCA AAG ACC CC	
	dsDNA-c	GGG GTC TTT GCT CAG CGC CAA CTA GAT CGA CAA GT	
ssDNA (Figure 2C)		ACT TGT CGA TCT AGT TGG CGC TGA GCA AAG ACC CC	Cy5
Primer (“6”)		CAAACG	

Table S2. A brief comparison of the method with former ones.

Name	Principle	Target molecule	LOD	*Cis*- and *trans*-cleavage	Time	Ref
The method	Target recycling+ cis- and trans-cleavage of Cas12a/crRNA	PBP2a protein	2.5 cfu/mL	Both	90 min	
Dual recognition	RCA+ trans-cleavage of Cas12a/crRNA	Protein A+ PBP2a	100 cfu/mL	*Trans*-cleavage	150 min	[1]
Dual modes	Exo-III enzyme assisted	*mecA* gene	57 cfu/mL	*Trans*-cleavage	120 min	[2]
Cas12a/crRNA-Based Biosensor	DNA polymerase assisted chain displacement	*mecA* gene	212 aM	*Trans*-cleavage	90 min	[3]
Colorimetric	CRISPR/Cas12a system and RPA	8	8 cfu/mL	*Trans*-cleavage	100 min	[4]

Notes: RPA, recombinase polymerase amplification; RCA, rolling circle amplification; LOD, limit of detection; Exo-III, exonuclease-III.

References:

1. Xu L, Dai Q, Shi Z, Liu X, Gao L, Wang Z*, et al.* 2020. Accurate MRSA identification through dual-functional aptamer and CRISPR-Cas12a assisted rolling circle amplification. *J. Microbiol. Methods.* **173:** 105917.
2. Xu J, Ma Q, Kang Y. 2025. Dual Mode Analysis of Methicillin-Resistant Staphylococcus aureus by the CRISPR/Cas12a-Assisted Exonuclease-Mediated Signal Cycle. *ACS Omega.* **10:** 17820-17826.
3. Lai F, Xia K, Lin W, Jian F, Yang H. 2024. CRISPR/Cas12a-Based APE1 Enzyme Cleavage Assay for Drug Resistance Analysis of Staphylococcus aureus-Related Pneumonia. *ACS Omega.* **9:** 31166-31172.
4. Wei L, Wang Z, Wang J, Wang X, Chen Y. 2022. Aptamer-based colorimetric detection of methicillin-resistant Staphylococcus aureus by using a CRISPR/Cas12a system and recombinase polymerase amplification. *Anal. Chim. Acta.* **1230:** 340357.
